# Human trafficking is more than a crime

**DOI:** 10.1016/j.lanwpc.2022.100444

**Published:** 2022-03-30

**Authors:** The Lancet Regional Health – Western Pacific

In late January, 2022, a video of a woman chained by her neck in Xuzhou village went viral on social media in China. The experience of this chained mother of eight children sparked enormous public anger and raised public demand for her rescue leading to an outpouring of debates about human trafficking in China among the public.

Human trafficking can happen in any age group and has many forms, but women and children are more susceptible to the crime and are disproportionally affected. One of the most common forms of human trafficking is sexual exploitation. Given the hidden nature and lesser reporting of such crimes, statistics and data for the prevalence of human trafficking is not always accurate. In 2016, the International Labour Office estimated that 40 million people were subjected to human trafficking, of whom 62% came from the Asia-Pacific region.

Gender discrimination, poverty, and migration status are the main factors that push victims into the trap of the trafficker. Numerous anti-trafficking interventions have been implemented at the international, regional, and local levels. The common forms of interventions are strengthening legislation, supporting survivors, and raising awareness campaigns, which line up with the anti-trafficking “3 P” framework of prosecution, protection, and prevention. In a systematic review including 90 reports on anti-trafficking programmes conducted between 2000 and 2015, Katharine Bryant and Todd Landman concluded that solid evidence on what works to prevent human trafficking is still lacking. There are some lessons learnt from years of intervention projects: the effect of campaigns to raise awareness is inadequate if not delivered with a clear message to a targeted community; legislation shows impacts but needs other key stakeholders to be involved in the process; and trauma-informed care and victim-centred assistance are crucial to support survivors. Instead of interventions targeting a single segment of human trafficking networks, we need an integrated approach that spans the entire trafficking chain from recruitment and transportation to exploitation. The integrated approach should also address environmental, social, and individual factors to proactively reduce risk of trafficking.

The health-care system has an important role to play in creating a safe and responsive environment for those subjected to trafficking. The exploitation and abuse during trafficking results in multiple and complex health problems, including injuries from violence, sexually transmitted infections, and adverse reproductive health outcomes. Survivors of human trafficking can experience anxiety, depression, and post-traumatic stress disorder. Among members of the public, health-care workers have an increased possibility of interacting with a potential victim. Therefore, it is essential that health-care professionals are properly trained to identify those who are being subjected to human trafficking as well as survivors, and to build trust within the health-care system for appropriate treatment and recovery. In this instance some progress has been made, for example, the USA has passed the Stop, Observe, Ask and Response (SOAR) to Health and Wellness Act of 2018 to provide health-care professionals with training in identifying and treating those subjected to trafficking. The International Organization for Migration has published a handbook called Caring for Trafficked Persons: Guidance for Health Providers to provide more detailed guidelines for international health-care providers to diagnose and treat people who have been trafficked. In the Western Pacific region, we need more engagement from health providers to develop tailored strategies and specific guidelines for the region, but the priority should begin with training them how to identify and treat potential victims in health-care settings.

Human trafficking is a not only a crime but also a public health problem that affects individuals and communities for generations. The fight against human trafficking cannot be tackled by one factor or by focusing on a single aspect of the problem. Other than the governments’ commitment to implementing anti-trafficking laws and to criminalising the traffickers, it is essential for the health-care system to be involved in trafficking prevention, victim identification, providing trauma-informed emergency and medical care, and helping survivors in their physical, psychological, and social recovery so they are less susceptible to trafficking in the future.

After being rescued from trafficking and admitted to a mental health hospital, the woman said, “the world doesn't want me”. We need to do more to prevent tragedies like this from happening.

